# The influence of metabolic energy on neural computation

**DOI:** 10.1186/1471-2202-14-S1-A2

**Published:** 2013-07-08

**Authors:** Simon Laughlin

**Affiliations:** 1 Department of Zoology, University of Cambridge, Cambridge CB2 3EJ, UK

## 

Computational Neuroscience is a vital part of the brave effort to reverse engineer brains, ultimately our own. Our efforts are confounded by an embarrassment of riches. Brains' winning technology, cell and molecular biology, enables neurons to connect and perform a huge variety of operations and adapt them with unparalleled ingenuity and subtly. Faced with so much that can be done, how do we discover what is done? Three constraints can guide us. One is what has to be done, the nature of the task and the operations that must be performed to generate the behavior that is observed. Another is data (usually incomplete) about what is being done. I will talk about the third constraint, physical, chemical and biological limits to what can be done and, in particular, energy consumption.

Beyond the realms of quantum computers, computation dissipates energy. Consequently energy supply and heat loss ultimately limit processing power. Here the brain is severely limited by its winning technology; neurons are low energy density devices and this restricts bandwidth and noise. I will discuss how brains' attempt to operate effectively with feeble neurons influences its unique style of computation, by considering chemical and electrical protein circuits, matching and adapting components, hybrid processing, redundancy reduction and its opposite, sparsification. I will propose that the efficient brain behaves like the Physics PhD Student from Hell, who does everything as slowly as possible, as inaccurately as possible and, wherever possible, uses chemistry. But, like many clever students, the brain is charmingly adaptable.

